# Beyond the abdomen: an unexpected finding of ectopic pancreas in a mediastinal mass

**DOI:** 10.1093/jscr/rjaf505

**Published:** 2025-08-12

**Authors:** William Sebastian, Ameer Metwally, Michelle Abramova, Thomas Bauer, Rachel NeMoyer

**Affiliations:** Department of Surgery at Jersey Shore University Medical Center (JSUMC), 1945 Route 33, Neptune, NJ 07753, United States; Department of Surgery at Jersey Shore University Medical Center (JSUMC), 1945 Route 33, Neptune, NJ 07753, United States; Department of Surgery at Jersey Shore University Medical Center (JSUMC), 1945 Route 33, Neptune, NJ 07753, United States; Department of Thoracic Surgery at Jersey Shore University Medical Center (JSUMC), 1945 Route 33, Neptune, NJ 07753, United States; Department of Thoracic Surgery at Jersey Shore University Medical Center (JSUMC), 1945 Route 33, Neptune, NJ 07753, United States

**Keywords:** ectopic pancreas, mediastinal mass, VATS

## Abstract

Ectopic pancreas (EP) is a rare congenital anomaly defined as pancreatic tissue lacking anatomical or vascular continuity with the pancreas. Although it occurs in 0.25%–2% of the population, mediastinal EP is exceedingly rare, with fewer than 30 reported cases. We present a case of a 51-year-old woman found to have a large anterior mediastinal mass incidentally during trauma workup. Imaging revealed a cystic lesion with rim calcification, concerning for teratoma. Surgical resection via sternotomy was performed due to size and complexity. Histopathology demonstrated a duplication cyst containing pancreatic acini, ducts, and islets—confirming mediastinal EP. The patient remained asymptomatic postoperatively. This case highlights the diagnostic challenges of mediastinal EP, which often mimics other anterior mediastinal masses and typically requires surgical resection for definitive diagnosis. Recognition of EP, even in asymptomatic patients, is critical to guide management and prevent misdiagnosis of potentially benign lesions.

## Introduction

Ectopic pancreas (EP) is defined as pancreatic tissue located outside its normal anatomical position, without any direct anatomical or vascular connection to the orthotopic pancreas [1]. Its reported prevalence is low—between 0.25% and 2%—and it is most often discovered incidentally at time of autopsy [2, 3]. EP is predominantly located within the gastrointestinal tract (most commonly in the stomach, small intestine, or within a Meckel’s diverticulum); accounting for 70%–90% of cases, but it can also be found in other intra-abdominal organs such as the liver or spleen, and in exceedingly rare cases at extra-abdominal sites [4, 5]. We present a case of an anterior mediastinum EP that was found incidentally in an asymptomatic patient following workup for a traumatic injury.

## Case

**Figure 1:**
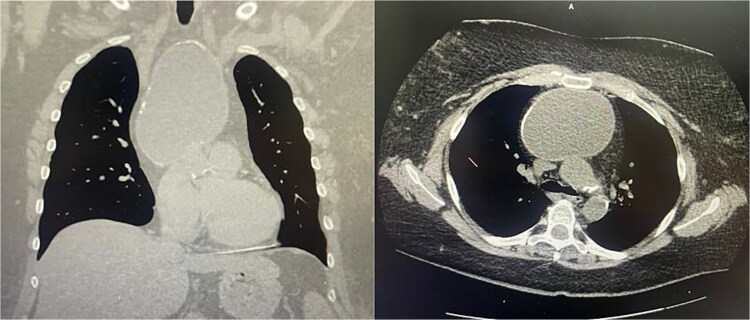
Coronal and axial views of a non-contrast CT of the chest demonstrating a large fluid density in the anterior mediastinum with rim calcification, subtle septations, and some nodularity, measuring 7.1 × 9.2 × 11.2 cm, accompanied by multiple small bilateral pulmonary nodules.

**Figure 2:**
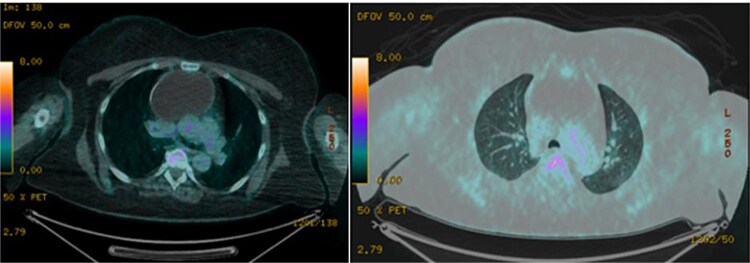
Axial images of PET/CT demonstrating a photopenic mass in the anterior mediastinum measuring 7.0 × 9.4 cm.

A 51-year-old woman with a history of insulin-dependent type 2 diabetes mellitus and Henoch-Schönlein purpura without history of tobacco or alcohol use presented to the emergency department after being struck by a slow-moving motor vehicle as a pedestrian. On initial evaluation, the patient reported mild shoulder pain but denied any further injuries or symptoms. Workup included a chest X-ray, which demonstrated mediastinal widening suggestive of thoracic aortic ectasia. Non-contrast computed tomography (CT) of the chest revealed a large fluid density in the anterior mediastinum with rim calcification, subtle septations, and some nodularity, measuring 7.1 × 9.2 × 11.2 cm, accompanied by multiple small bilateral pulmonary nodules (Fig. 1). The radiologist was concerned about a possible cystic teratoma. A subsequent positron emission tomography (PET-CT) scan confirmed a photopenic mass in the anterior mediastinum measuring 7.0 × 9.4 cm, with no evidence of regional lymphadenopathy (Fig. 2). The patient remained asymptomatic and elected to undergo surgical resection.

Surgical planning called for a right-sided, robotic-assisted thoracoscopic surgery (RATS). Intraoperatively, the mass was carefully dissected away from the right phrenic nerve, pericardium, and internal mammary vessels. Dense adhesions and the inability to progress safely due to limited visibility superiorly necessitated conversion to an open median sternotomy, which was discussed preoperatively due to the large size of the tumor. Upon placement of the sternal retractor, the innominate vein was noted to be markedly compressed and adherent to the lesion; however, it was eventually freed from this vessel. The mass intermittently discharged thick, yellow fluid, which was aspirated and submitted for culture. After decompression, the mass was completely mobilized from the right internal thoracic artery, pericardium, and great vessels; however, a 2 × 3 cm segment adherent to the left phrenic nerve could not be safely detached and was left in situ after cauterization of the cystic cavity. The resected specimen measured 10.5 × 8 × 3 cm and was predominantly cystic.

Histopathological analysis demonstrated a multilocular cyst lined by bland, mostly denuded columnar epithelium containing cytoplasmic mucin. Immunohistochemical staining showed positivity for CK7 and CDX2 and negativity for CK20, consistent with an upper gastrointestinal or pancreaticobiliary epithelial phenotype. The cyst wall demonstrated ectopic pancreatic tissue composed of acini, ducts, and neuroendocrine islets positive for chromogranin and synaptophysin and also harbored parathyroid and thymic tissue within a fibrotic stroma. Cytological examination revealed no evidence of malignancy. At 6-month follow-up, the patient remained asymptomatic, and surveillance CT imaging demonstrated no obvious residual disease.

## Discussion

EP is a rare congenital anomaly, reported in ~2% of the general population. When present within the mediastinum, EP typically appears as part of a duplication cyst or a mature teratoma. In our case, histopathological examination revealed tissue arising exclusively from ectopic pancreatic tissue, making a duplication cyst more likely than a teratoma.

Interestingly, glandular epithelial cells were identified within the EP; however, no definitive evidence of endocrine or exocrine functional activity was observed. Although uncommon, there are documented cases of mediastinal EP exhibiting functional exocrine or endocrine behavior. One such case involved spontaneous rupture of the lesion due to autodigestion from active digestive enzymes [1]. Theoretically, increased endocrine function from an EP could contribute to systemic disease processes, such as diabetes mellitus, as seen in our case. Nonetheless, current literature lacks definitive evidence supporting this hypothesis.

Our case represents an exceptionally rare phenomenon: an EP forming a substantial anterior mediastinal mass measuring 10.5 × 8 × 3 cm. To date, only ~30 cases of mediastinal EP have been reported. While the majority of these cases presented with clinical symptoms—such as chest pain or hemoptysis—our patient was entirely asymptomatic, with the lesion discovered incidentally during imaging performed for trauma-related evaluation [6].

A cystic component was present in most reported mediastinal EP cases, consistent with our findings [6]. Diagnostic imaging typically begins with a chest X-ray, which may reveal an altered mediastinal silhouette or contour, followed by CT to further characterize the lesion. However, CT imaging alone cannot reliably distinguish EP from other anterior mediastinal masses, including thymoma, lymphoma, or teratoma. In most reported instances, surgical resection followed by histopathological evaluation has been necessary to establish a definitive diagnosis and remains the standard of care. Notably, there has been at least one reported case where mediastinal EP was diagnosed via endoscopic ultrasound (EUS) with fine-needle aspiration and managed conservatively without surgical intervention [7–9].
